# Supplementary material for: Implementing the Maternal Postnatal Attachment Scale (MPAS) in universal services: Qualitative interviews with health visitors
Source: Wellcome Open Res. 2023 Jan 6;7:85. Originally published 2022 Mar 11. [Version 2] doi: 10.12688/wellcomeopenres.17551.2 (PMC9975410; doi:10.12688/wellcomeopenres.17551.2)
Supplement: Peer review history

# Peer review: Reviewer response for version 2

**DOI:** 10.21956/wellcomeopenres.20361.r53968

**Published:** 2023-01-16

**Authors:** Trecia A. Wouldes

**Affiliations:** 1 Department of Psychological Medicine, University of Auckland, Auckland, New Zealand

I am happy to approve Version 2.

Is the work clearly and accurately presented and does it cite the current literature?

Yes

If applicable, is the statistical analysis and its interpretation appropriate?

Not applicable

Are all the source data underlying the results available to ensure full reproducibility?

Yes

Is the study design appropriate and is the work technically sound?

Yes

Are the conclusions drawn adequately supported by the results?

Yes

Are sufficient details of methods and analysis provided to allow replication by others?

Yes

Reviewer Expertise:

NA

I confirm that I have read this submission and believe that I have an appropriate level of expertise to confirm that it is of an acceptable scientific standard.

# Peer review: Reviewer response for version 1

**DOI:** 10.21956/wellcomeopenres.19408.r51479

**Published:** 2022-08-01

**Authors:** Trecia A. Wouldes

**Affiliations:** 1 Department of Psychological Medicine, University of Auckland, Auckland, New Zealand

This qualitative study aimed to determine the challenges and acceptability reported by health visitors of using a structured assessment of the parent-infant relationship (the Maternal Postnatal Attachment Scale, MPAS). Semi-structured interviews were conducted with 11 health visitors and data from transcripts were analysed using thematic analysis. The results of this study were then discussed in relation to a quantitative study evaluating the psychometric properties of the MPAS also submitted for publication by members of this research group (Dunn *et al.,* submitted).

 This was a very well-written article that provided some important information as to the challenges and acceptability of using the MPAS in clinical practice in an ethnically diverse, deprived population in the UK. Five themes were identified by the two researchers who independently coded the transcripts (PB and ZH). These included the importance the health visitors placed on evaluating the parent-infant relationship; concerns around the method of evaluating the parent-infant relationship (questionnaire vs observation); the benefits of the MPAS, especially in promoting a discussion around any concerns; the challenges of using a structured questionnaire during health visits; and recommendations of how the MPAS could be improved.

 As in any qualitative study, the lens through which the data is evaluated is important, therefore, it would be useful to know the clinical and/or research background of the researchers who interpreted the data. Lacking also was a more in-depth description of the health visitors' ethnicity and background, other than simply their gender. As the authors reported, this was an ethnically diverse area so the ethnicity of the health visitors would be of particular importance.

 Finally, as the discussion largely focused on using the quantitative and qualitative research to consider the clinical utility of the MPAS, it would be important to have access to both in evaluating the conclusions made by these authors.

Is the work clearly and accurately presented and does it cite the current literature?

Yes

If applicable, is the statistical analysis and its interpretation appropriate?

Not applicable

Are all the source data underlying the results available to ensure full reproducibility?

Yes

Is the study design appropriate and is the work technically sound?

Yes

Are the conclusions drawn adequately supported by the results?

Yes

Are sufficient details of methods and analysis provided to allow replication by others?

Yes

Reviewer Expertise:

I am a developmental scientist. My research methods are predominantly prospective, longitudinal research with high-risk, ethnically diverse populations. Infant mental health and parent-infant relationships of high-risk families are a focus of my research, teaching and policy work.

I confirm that I have read this submission and believe that I have an appropriate level of expertise to confirm that it is of an acceptable scientific standard, however I have significant reservations, as outlined above.

# Response

**Published:** 2022-11-03

**Authors:** Charlotte Endacott

**Affiliations:** Born in Bradford, UK

**Reviewer comment:** This was a very well-written article that provided some important information as to the challenges and acceptability of using the MPAS in clinical practice in an ethnically diverse, deprived population in the UK. Five themes were identified by the two researchers who independently coded the transcripts (PB and ZH). These included the importance the health visitors placed on evaluating the parent-infant relationship; concerns around the method of evaluating the parent-infant relationship (questionnaire vs observation); the benefits of the MPAS, especially in promoting a discussion around any concerns; the challenges of using a structured questionnaire during health visits; and recommendations of how the MPAS could be improved.

**Author response:** We would like to thank the reviewer for their helpful review.

**Reviewer comment:** As in any qualitative study, the lens through which the data is evaluated is important, therefore, it would be useful to know the clinical and/or research background of the researchers who interpreted the data. Lacking also was a more in-depth description of the health visitors' ethnicity and background, other than simply their gender. As the authors reported, this was an ethnically diverse area so the ethnicity of the health visitors would be of particular importance.

**Author response:** This is a useful point. We have added some information to the methods section: *The researchers had different academic backgrounds: public health (PB), anthropology (AC) and education studies (with early childhood) (ZH). As this may have shaped interpretation and identification of themes, this approach was adopted* *to improve completeness and include different perspectives.*

 It would have been very helpful to include discussion of the role of health visitor backgrounds and ethnicity. Unfortunately we did not ask participants to identify their background and ethnicity in the interviews. We would only want to report ethnicity as identified by participants themselves. We also had some concerns that reporting ethnicity could lead to identification of health visitors. However, this is an important point for us for us to consider for future research. We have therefore added this as a limitation:

*Second, we are unable to consider the role of health visitor backgrounds or ethnicity in their responses. Health visitors provided some background information during interviews, but we did not ask all health visitors to identify their ethnicity. It has become clear that this information could have been helpful to improve understanding and should be collected and reported carefully to protect anonymity in future research in this area.*

**Reviewer comment:** Finally, as the discussion largely focused on using the quantitative and qualitative research to consider the clinical utility of the MPAS, it would be important to have access to both in evaluating the conclusions made by these authors.

**Author response:** We agree that it is helpful to be able to read both the qualitative and quantitative papers together. The quantitative evaluation of the feasibility of MPAS in health visiting practice (Dunn et al 2022) is now published and available here: https://wellcomeopenresearch.org/articles/7-88

 We have also updated this reference throughout the paper.

# Peer review: Reviewer response for version 1

**DOI:** 10.21956/wellcomeopenres.19408.r49771

**Published:** 2022-04-26

**Authors:** Sharin Baldwin

**Affiliations:** 1 Warwick Clinical Trials Unit, University of Warwick, Warwick, UK; 2 London North West NHS Healthcare Trust, Harrow, UK

This is a well written paper on an important area of research. This paper discusses the implementation of the Maternal Postnatal Attachment Scale (MPAS) within the health visiting service in Bradford, used with mothers 3-4 months after birth. Eleven health visitors volunteered to participate in qualitative interviews and while a small number of health visitors thought the scale to be a useful tool, a number of challenges/ barriers were identified. This included language, interpretation of the questions, time constraints and lack of services to refer to following assessment.

 While the authors stated that all health visitors were trained and used the MPAS, it would be good to have more detail, such as: how many were trained in total, how many used them fully, how many used them partially, and how many did not use them at all? Also, is there any data on how many mothers would have been eligible to be assessed using MPAS during that 6 month period, and how many were actually assessed using MPAS? Another aspect that remains unclear is that do all mothers receive a visit at 3-4 months? If not, what proportion do and why was this period chosen for the MPAS assessment? It would be good to mention fathers too and whether there is a need to have a tool that assesses parental-child interaction/attachment rather than just maternal, given that many fathers may be the main carer to their child or there could be both parents who are male. 

 Overall, I feel this is a very interesting article that would be of interest to health visitors and other health professionals working with parents and children.

Is the work clearly and accurately presented and does it cite the current literature?

Yes

If applicable, is the statistical analysis and its interpretation appropriate?

Not applicable

Are all the source data underlying the results available to ensure full reproducibility?

Yes

Is the study design appropriate and is the work technically sound?

Yes

Are the conclusions drawn adequately supported by the results?

Yes

Are sufficient details of methods and analysis provided to allow replication by others?

Yes

Reviewer Expertise:

Fathers' mental health, Perinatal mental health, Health visiting, evaluation of complex interventions, Mixed methods research

I confirm that I have read this submission and believe that I have an appropriate level of expertise to confirm that it is of an acceptable scientific standard.

# Response

**Published:** 2022-11-03

**Authors:** Charlotte Endacott

**Affiliations:** Born in Bradford, UK

**Reviewer comment:** This is a well written paper on an important area of research. This paper discusses the implementation of the Maternal Postnatal Attachment Scale (MPAS) within the health visiting service in Bradford, used with mothers 3-4 months after birth. Eleven health visitors volunteered to participate in qualitative interviews and while a small number of health visitors thought the scale to be a useful tool, a number of challenges/ barriers were identified. This included language, interpretation of the questions, time constraints and lack of services to refer to following assessment.

**Author response:** We would like to thank the reviewer for their helpful comments.

**Reviewer comment:** While the authors stated that all health visitors were trained and used the MPAS, it would be good to have more detail, such as: how many were trained in total, how many used them fully, how many used them partially, and how many did not use them at all? Also, is there any data on how many mothers would have been eligible to be assessed using MPAS during that 6 month period, and how many were actually assessed using MPAS?

**Author response**: We decided to present the quantitative information on use of the MPAS in the linked paper by Dunn et al, which has now been published in Wellcome Open Research here: https://wellcomeopenresearch.org/articles/7-88 . This includes quantitative information on the number of health visitors who used the MPAS, the number of women eligible and number who completed the MPAS assessment. We felt that it was better to present the quantitative and qualitative information in separate papers so that we were able to go into detail. 

 We have clarified this in paragraph at the end of the introduction:

*The first (* *Dunn et al., 2022* *) presented an introduction to MPAS and quantitative findings on the measure’s psychometric properties and the number of health visitors who used the tool, the number of eligible women and rates of completion over the study period. Findings showed that the tool has limited validity for assessing mothers’ affective bond to their infant, suggesting that it is not robust enough to recommend ongoing use in the Bradford context. During the study period, 95% of health visitors working in the pilot area used the tool, and MPAS was completed with 37% of eligible women in the sample.*

**Reviewer comment:** Another aspect that remains unclear is that do all mothers receive a visit at 3-4 months? If not, what proportion do and why was this period chosen for the MPAS assessment?

**Author response:** We have added information to the introduction after explanation about why the MPAS tool was chosen:

*The 3–4 month health visitor contact is offered as an additional universal contact by health visiting in Bradford (it is not nationally mandated) and was identified as a clinically useful and feasible time-point for assessment.*

 Quantative information on health visitor contacts at 3-4 months is provided in the linked paper (Dunn et al).

**Reviewer comment:** It would be good to mention fathers too and whether there is a need to have a tool that assesses parental-child interaction/attachment rather than just maternal, given that many fathers may be the main carer to their child or there could be both parents who are male. 

**Author response:** We agree that this would be very important – there is a lack of understanding of paternal attachment and mental health. There is an equivalent tool for fathers – the Paternal Postnatal Attachment Scale (PPAS), however unfortunately there are no universal health checks with fathers after birth in the UK, so it was not feasible to pilot this as part of this study. We were able to include a measure of attachment in the questionnaire that partners complete during pregnancy as part of the BiBBS cohort (Dickerson et al 2016 https://bmcpublichealth.biomedcentral.com/articles/10.1186/s12889-016-3318-0).

**Reviewer comment:** Overall, I feel this is a very interesting article that would be of interest to health visitors and other health professionals working with parents and children.

**Author response:** Thank you again for your helpful comments.
